# *Helicobacter pylori* CagL Y58/E59 Mutation Turns-Off Type IV Secretion-Dependent Delivery of CagA into Host Cells

**DOI:** 10.1371/journal.pone.0097782

**Published:** 2014-06-03

**Authors:** Nicole Tegtmeyer, Judith Lind, Benedikt Schmid, Steffen Backert

**Affiliations:** 1 Friedrich Alexander University Erlangen, Department of Biology, Division of Microbiology, Erlangen, Germany; 2 Friedrich Alexander University Erlangen, Department of Biology, Division of Biotechnique, Erlangen, Germany; Rush University Medical Center, United States of America

## Abstract

The type IV secretion system (T4SS) is a major virulence determinant of the gastric pathogen *Helicobacter pylori.* The CagL protein is a specialized adhesin of the corresponding T4SS pilus, which establishes initial contact with the integrin β1 receptor on host target cells. Recent studies proposed that Y58 and E59 amino acid polymorphisms in CagL increase the virulence of *H. pylori* strains by enhanced translocation and phosphorylation of the CagA effector protein. These polymorphisms were therefore correlated with an increased risk of gastric cancer development. Here we show that the Y58/E59 motif, which is located in a loop connecting two α-helices, and corresponding polymorphisms could influence the function of CagL. However, expression of isogenic CagL Y58/E59 variants in *H. pylori* strain 26695 significantly blocked the translocation and phosphorylation of CagA as compared to complemented wild-type CagL. These results suggest that the function of the T4SS for delivery of CagA is turned-off by the Y58/E59 mutation in CagL. This activity appears to be similar to the one recently described for another T4SS pilus protein, CagY, which is also sufficient to cause gain or loss of T4SS function. These data support the hypothesis that certain mutations in CagL or recombination events in CagY may serve as a sort of molecular switch or perhaps rheostat in the T4SS, which could alter the function of the pilus and "tunes" injection of CagA and host pro-inflammatory responses, respectively.

## Introduction

In this Formal Comment we refer to the recent publication in PLOS ONE by Yeh and co-workers [1]. This report claims that the *Helicobacter pylori* CagL amino acid polymorphisms Y58 and E59 increase the virulence of corresponding clinical strains. CagL is a specialized adhesin encoded by the *cag* pathogenicity island (*cag*PAI). During infection, CagL is recruited to the surface of a type IV secretion system (T4SS) pilus structure, mediating contact with the integrin α5β1 receptor and translocation of the virulence factor CagA [2]. Yeh and co-workers produced *H. pylori* CagL mutants in one strain (Hp1033) followed by infection of AGS gastric epithelial cells and investigated the expression of integrin α5β1, CagA translocation/phosphorylation and other parameters [1]. It was claimed that *H. pylori* CagL Y58/E59 point mutants retain active integrin β1 with stronger binding affinity and significantly enhance CagA translocation and phosphorylation as compared to wild-type CagL. The above study was based on an earlier publication by the same group, reporting that CagL sequence polymorphisms in *H. pylori* correlated with clinico-histological outcomes and gastric α5β1 integrin expression [3]. In this study, 145 patients with *H. pylori* infection and different gastric diseases were investigated. The isolates from the gastric cancer (GC) group of patients revealed a higher rate of CagL Y58/E59 amino acid sequence polymorphisms than those in non-GC patients (*P < 0.05*). The authors therefore correlated the Y58/E59 polymorphisms in CagL with an increased risk of GC development [3], which was now interpreted to arise by higher binding capacities of CagL to integrin α5β1 and enhanced injection of CagA into gastric epithelial cells [1].

**Figure 1:**
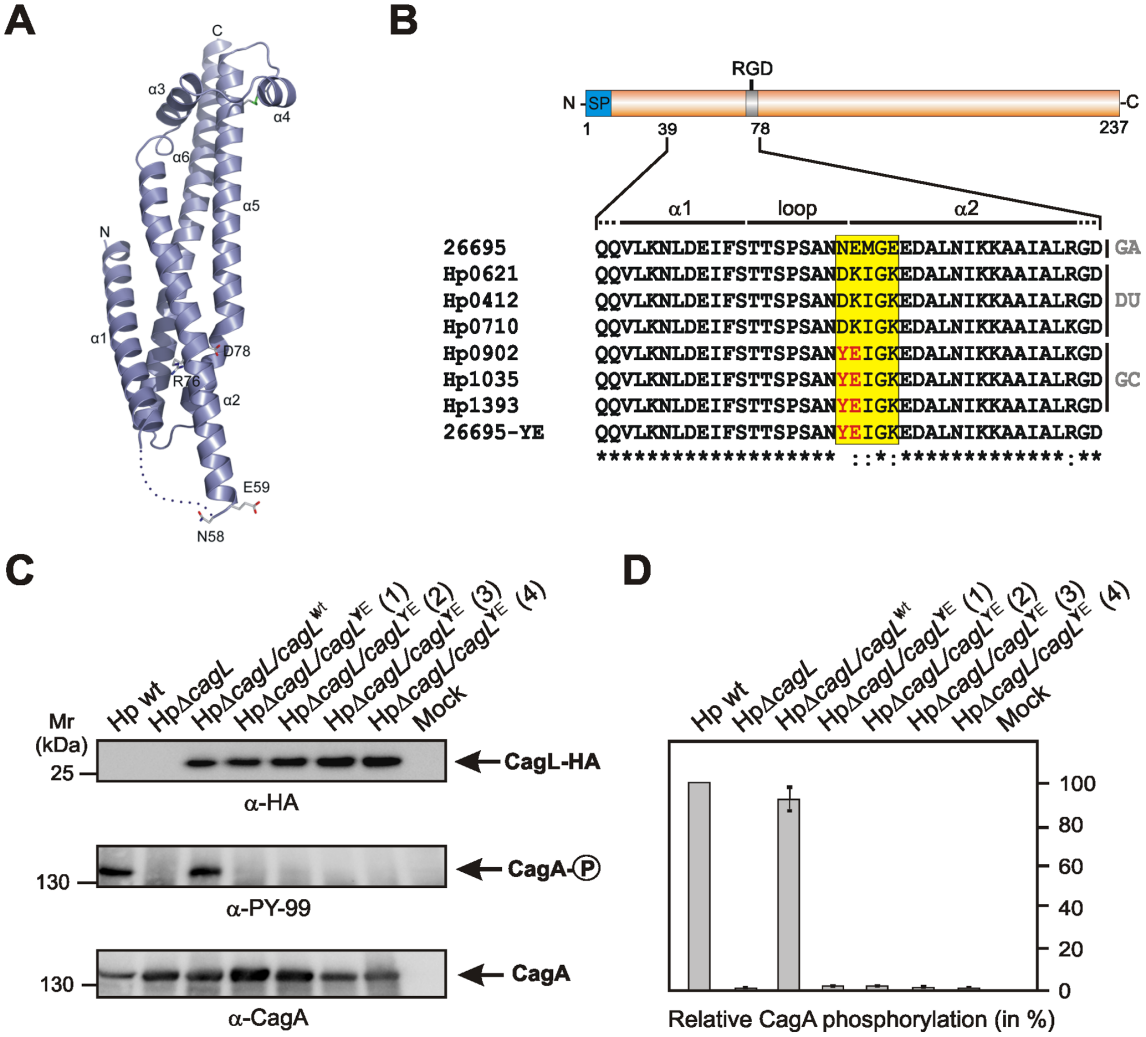
Role of *Helicobacter pylori* CagL Y58/E59 mutation in type IV secretion-dependent delivery of CagA in host cells. (**A**) Cartoon representation of the crystal structure of CagL (molecule A of the six crystallographically independent molecules, PDB accession code 3ZCJ) from strain 26695 [12]. The various α helices (denoted 1–6), R76 and D78 of the RGD-motif and the amino acids N58 und E59 are highlighted. The amino acids from T52-N57 are not resolved and are shown as a dashed line. This region connects the two indicated helices, α1 (D22-S50) and α2 (E59-K101). The PyMOL program was used to generate the structure illustration (The PyMOL Molecular Graphics System, Schrödinger, LLC). (**B**) Alignment of amino acid sequences of CagL (position 39–78) among strains 26695 [2], Hp0621, Hp0412, Hp0710, Hp0902, Hp1035, Hp1393 [3] and the 26695 CagL^YE^ mutant produced here is shown. A variable region among the CagL proteins between positions 58–62 is boxed in yellow and the Y58/E59 polymorphism is highlighted with red. The strains originated from gastritis (GA), duodenal ulcer (DU) and gastric cancer (GC) patients as indicated [3]. (**C**) AGS gastric epithelial cells were infected with the indicated *H. pylori* strains and *cagL* mutants for 8 hours. Resulting protein lysates were probed with the indicated antibodies as described. The experiments were done at pH 7.4, which gave very strong phospho-CagA signals in a recent study [1]. (**D**) Quantification of CagA phosphorylation signals in panel C using the luminescence image analyzer. The strongest signal in lane 1 was set as 100%. The data are representative from three independent experiments.

We have previously reported that CagL contains an RGD-motif, like the human extracellular matrix protein fibronectin, and is able to trigger RGD-dependent binding to integrin α5β1 during infection [2]. It was also shown that purified CagL alone, in an RGD-dependent fashion, can directly induce intracellular signaling pathways upon contact with mammalian cells including kinase activation [4], gastric acid suppression [5], [6], β-defensin-1 suppression [7] and production of IL-8 [8]. During interaction with various human and mouse cell lines, CagL mimics fibronectin in triggering cell spreading, focal adhesion formation, and activation of several tyrosine kinases in an RGD-dependent manner. Among the activated factors are the kinases FAK and Src, but also the actin-binding protein cortactin and EGF receptor members EGFR and Her3/ErbB3 [4], [5], [9]. It was also demonstrated that CagL can interact with the integrin member αvβ3 with yet unknown consequences for the host cell [10]. In addition, CagL can bind to the integrin member αvβ5 to induce signaling leading to gastrin production in an RGD-independent manner [11]. Very recently, we presented the crystal structure of CagL revealing an elongated four-helix bundle [12], [13]. The RGD-motif is surface-exposed but located within a long α helix (Figure 1A,B), which is unprecedented as previously characterized integrin-binding RGD-motifs are located within extended or flexible loops [12]. Comparison of seven crystallographically-independent CagL molecules revealed substantial structural flexibility, leading to the hypothesis that CagL may partly unfold during receptor binding [12]. Here we aimed to investigate the role of Y58/E59 amino acid polymorphisms in CagL by mutagenesis and infection experiments.

## Results

After the first publication by Yeh and co-workers in 2011 [3, we got interested in the potential role of CagL Y58/E59 polymorphisms in host cell interactions by the *H. pylori* T4SS. The CagL crystal structure reveals that the amino acids at position 58 and 59 are located at the end of a loop between two neighboring helices, α1 and α2 (Figure 1A/B). Considering the structural flexibility in this region [12], we assumed that mutation to the Y58/E59 residues may cause a change in CagL structure, leading to altered integrin interaction which could influence CagA translocation. To investigate this hypothesis, we first deleted the entire *cagL* gene in the *cag*PAI of *H. pylori* and complemented wild-type *cagL* gene of strain 26695 in the urease gene locus using a so-called ‘double cross-over’ construct as described [9],[14]. While wild-type *H. pylori* was able to produce phosphorylated CagA in infected AGS cells, the *cagL* deletion mutant did not as expected (Figure 1C/D and Figure S1). Complementation of the *cagL* mutant strain with wild-type *cagL* carrying a hemagglutinin (HA)-tag restored CagA translocation to wild-type levels, indicating that our complementation approach works (Figure 1C/D and Figure S1). We have then noted that the CagL Y58/E59 region of the used GC strains contains some additional amino acid exchanges at position 60–62 [3] (Figure 1B, yellow box). We therefore introduced the amino acids 58–62 of GC strains in CagL of strain 26695 in order to generate a CagL Y58/E59 expressing mutant, CagL^YE^ (Figure 1B, bottom). We have collected four different clones, which were confirmed by PCR and standard sequencing, indicating that the mutation was introduced correctly into *H. pylori*. As a further control, we monitored the expression of the 26 kDa CagL protein using α-HA antibodies. The results show that CagL is produced in similar amounts between the complemented wild-type and Y58/E59 expressing mutants (Figure 1C). However, to our great surprise, infection experiments have shown that the CagL^YE^ variant completely suppressed the production of phosphorylated CagA as compared to complemented wild-type CagL as monitored in a time course of 1–8 hours (Figure 1C and data not shown). Three different experiments with four individual CagL^YE^ clones (1–4) gave the same results (Figure 1C/D). Thus, our experiments revealed entirely different data to those reported by Yeh and co-workers [1].

## Discussion

CagL is one of the most studied factors of the *H. pylori* T4SS encoded by the *cag*PAI [15]–[18]. We show here that expression of an isogenic CagL Y58/E59 variant in *H. pylori* strain 26695 significantly blocked the translocation and phosphorylation of CagA as compared to complemented wild-type CagL. These data are in contrast to a recently published paper [1]. As a possible explanation for the conflicting data, we cannot exclude the possibility of specific genetic differences between strains 26695 and Hp1033. However, we have noted that Yeh and co-workers performed a very unusual procedure to introduce point mutants in *H. pylori* [1]. The *cagL* gene was first disrupted by introduction of a chloramphenicol resistance (Chl^R^) cassette as selective marker. Thus, the *cagL* gene was not deleted from the chromosome, which offers the possibility of unwanted ‘single cross-over’ events during transformation. It is well known that recombination in *H. pylori* by ‘single cross-over’ leads to integration of the entire plasmid into the chromosome [19], with the result that two copies of *cagL* genes will appear in the transformants. This would mean that both the *cagL* wild-type and *cagL* mutant are possibly present in the chromosome, and both genes can easily recombine with each other during cultivation. However, to generate Y58/E59 mutants, this clone was then transformed with the *cagL* gene carrying the various mutations, and the authors screened for one clone that lost the Chl^R^ cassette [1]. Since chromosomal transformation rates in *H. pylori* are commonly between 10^−5^ to 10^−6^ per microgram DNA [20], this would indicate that Yeh and co-workers must have screened at least 100,000 colonies per experiment in order to get one Chl^R^–negative clone. This experimental design is technically extremely difficult and was not described. However, it also remained unclear how correct introduction of the Y58/E59 mutations and exclusion of wild-type *cagL* gene presence in the Hp1033 chromosome has been confirmed [1]. In addition, the sequence of wild-type *cagL* from strain Hp1033 is not provided by the authors nor deposited in a gene database, and *cagL* expression was not tested by Western blotting or RT-PCR [1]. It is therefore very likely that the generated mutant clone can still express functional CagL wild-type protein, which could explain their results. By comparison, we excluded ‘single cross-over’ events by deleting entire *cagL* from the chromosome before introducing the *cagL* mutant allele [9],[14]. In addition, we have confirmed our *cagL* mutagenesis and correct expression by several independent approaches including PCR, sequencing and Western blotting. We also tested various individual *cagL* YE mutant clones, which always gave the same results as described above. We therefore suggest that the function of the T4SS for delivery of CagA is turned-down by the Y58/E59 mutation in CagL of 26695 and does not enhance T4SS functions as claimed by Yeh and co-workers [1].

We think that the above example of contradictory data underlines the important necessity that extreme care should be taken when performing mutagenesis in *H. pylori*, which is the proper basis for subsequent functional studies. Based on the above results, our Formal Comment offers putative reasons for the conflicting data and should help to diminish uncertainty in the scientific community. Interestingly, another recent report demonstrated in murine and non-human primate models that immune-driven host selection for recombination in another *H. pylori cag*PAI protein, CagY, is also sufficient to cause gain or loss of T4SS function with regard to IL-8 induction [21]. These data together support the hypothesis that certain variations in both proteins, CagY and CagL, may function as sort of molecular switches or perhaps rheostats in the T4SS which can alter the function of the pilus and "tunes" injection of CagA and host pro-inflammatory responses, respectively. In future experiments the function of such polymorphisms for the infection process should be studied in more detail using a large number of *H. pylori* strains from patients with different disease outcome.

## Materials And Methods

### Ags Cell Culture

The human gastric adenocarcinoma cell line AGS (ATCC CRL-1739™) was cultivated in RPMI 1640 medium, which was supplemented with 10% fetal calf serum (Gibco, Paisley, UK). Cells were grown at 37°C and 5.0% (v/v) CO_2_ and subcultivated in a ratio of 1∶3–1∶5 every 2–3days at a confluence of 70% to 80%.

### *H. Pylori* Strains And Infection Studies

We are using *cagL* of the fully sequenced strain 26695 as a model (accession number NC_000915). The *H. pylori* wild-type strain and isogenic *Hp*Δ*cagL* deletion mutant were generated and grown as described [14]. To complement the *Hp*Δ*cagL* mutant strain, the wild-type *cagL* gene was introduced into the chromosomal *ureA* locus, using a pAD1-derived plasmid [14]. CagL proteins expressed from the *ureA* promoter contain a hemagglutinin (HA) tag introduced following the signal sequence at amino acid position 22 [14]. For infection experiments, *H. pylori* were grown for 2days in thin layers and added at a multiplicity of infection (MOI) of 100 [22]. All experiments were done in triplicate.

### Site-Directed Mutagenesis Of Cagl

Site-directed mutagenesis of CagL was performed using the pAD1 vector as DNA template [9]. As shown in Figure 1B, CagL of GC strains contain the amino acids Tyr-Glu-Ile-Gly-Lys (YEIGK) at position 58–62 of the protein, while strain 26695 has the amino acids Asn-Glu-Met-Gly-Glu (NEMGE) at this position. To generate a CagL 26695^YE^ mutant (Figure 1B, bottom), we performed PCR reactions with the following primers: 628F 5′-GGTAAAGAAGATGCTCTAAACATC and 628R 5′-GATTTCATAATTAGCACTAGGGCTAG to open and amplify the entire construct. For amplification, Phusion^®^ High-Fidelity DNA Polymerase (NEB, Ipswich, USA) was used, followed by PCR purification (MinElute PCR Purification Kit, Qiagen, Hilden, Germany), digestion with *Dpn*I (Promega, Madison, USA), and ligation using T4 DNA Ligase (Promega). Re-sequencing and Western blotting of *E. coli* or *H. pylori* lysates, respectively, verified the appropriate expression of CagL mutant variants from the resulting plasmids.

### Antibodies And Western Blotting

Infected cells were harvested in ice-cold PBS containing 1 mM Na_3_VO_4_ (Sigma-Aldrich). Western blotting was done as previously described [22]. Rabbit α-CagL antiserum was raised against the C-terminal peptide (C-RSLEQSKRQYLQER) of the protein and was prepared by Biogenes (Berlin, Germany). The α-HA-tag antibody (NEB Cell Signaling, Frankfurt/M., Germany) was also used to detect tagged CagL. The pan-α-phosphotyrosine antibody PY-99 (Santa Cruz) and α-CagA (Austral Biologicals, San Ramon, CA, USA) were used to investigate the phosphorylation of CagA [23]. The α-glyceraldehyde-3-phosphate dehydrogenase (GAPDH) antibody (Santa Cruz) served as loading control in each Western blot (data not shown). As secondary antibodies, horseradish peroxidase conjugated α-mouse, α-rabbit, or α-goat polyvalent sheep immunoglobulin were used and antibody detection was performed with the ECL Plus chemiluminescence kit (Amersham Pharmacia Biotech) [24]. Band intensities were quantitated with the Lumi-Imager F1 (Roche Diagnostics, Mannheim, Germany) [25]. The data are representative from three independent experiments.

### Statistical Analysis

All data were evaluated using Student ***t***-test with SigmaStat statistical software (version 2.0). Statistical significance was defined by ***P***≤0.05 (*) and ***P***≤0.005 (**). All error bars shown in Fig. 1 and those quoted following the ± signs represent standard deviation.

## Supporting Information

Figure S1 **Role of *Helicobacter pylori* CagL Y58/E59 mutation in type IV secretion-dependent delivery of CagA in host cells.** AGS gastric epithelial cells were infected with the indicated *H. pylori* strains and *cagL* mutants for 8 hours using a multiplicity of infection of 100. Resulting protein lysates were probed with the indicated antibodies as described. This figure shows the original uncropped Western blots underlying Figure 1C. The cut sections are marked with boxes. The red asterisk in the α-PY-99 blot marks the phosphorylated 125 kDa host cell protein vinculin, which always runs below the phospho-CagA band at about 140 kDa [26].
(TIF)
