# Supplementary figures and images for: Helicobacter pylori CagL Y58/E59 Mutation Turns-Off Type IV Secretion-Dependent Delivery of CagA into Host Cells
Source: PLoS One. 2014 Jun 3;9(6):e97782. doi: 10.1371/journal.pone.0097782 (PMC4043526; doi:10.1371/journal.pone.0097782)

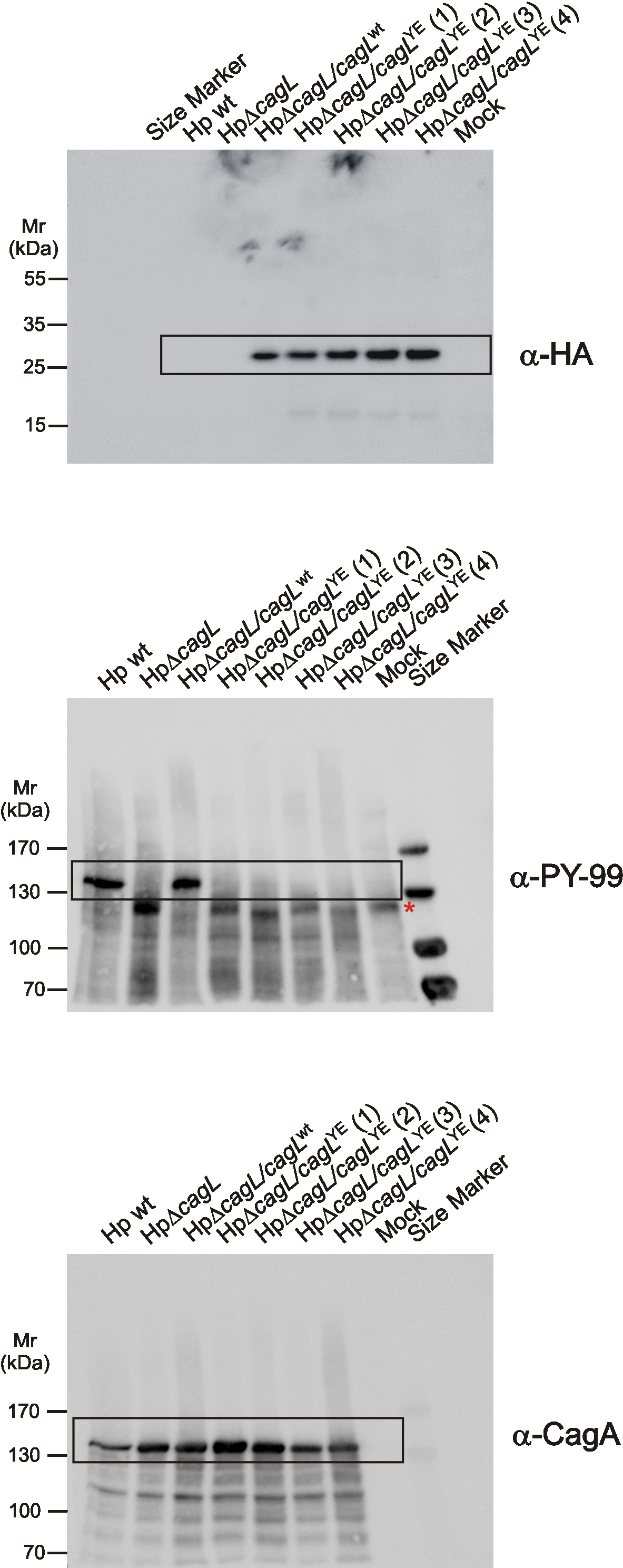

Supplement: Figure S1 — Role of Helicobacter pylori CagL Y58/E59 mutation in type IV secretion-dependent delivery of CagA in host cells. AGS gastric epithelial cells were infected with the indicated H. pylori strains and cagL mutants for 8 hours using a multiplicity of infection of 100. Resulting protein lysates were probed with the indicated antibodies as described. This figure shows the original uncropped Western blots underlying Figure 1C. The cut sections are marked with boxes. The red asterisk in the α-PY-99 blot marks the phosphorylated 125 kDa host cell protein vinculin, which always runs below the phospho-CagA band at about 140 kDa [26]. (TIF) [file pone.0097782.s001.tif]
